# MicroRNA-218 promotes early chondrogenesis of mesenchymal stem cells and inhibits later chondrocyte maturation

**DOI:** 10.1186/s12896-018-0496-0

**Published:** 2019-01-15

**Authors:** Song Chen, Zhenyu Xu, Jiahua Shao, Peiliang Fu, Haishan Wu

**Affiliations:** 1 Department of Arthroplasty Surgery, Changzheng Hospital, Second Military Medical University, Shanghai, 200003 China; 2 State Key Laboratory for Diagnosis and Treatment of Infectious Diseases, and Collaborative Innovation Center for Diagnosis and Treatment of Infectious Diseases, School of Medicine, Zhejiang University, Hangzhou, 310003 China

**Keywords:** Articular cartilage, microRNA-218, Mesenchymal stem cells, Chondrogenesis, Chondrocyte maturation

## Abstract

**Background:**

MicroRNAs (miRNAs) reportedly participate in the mesenchymal stem cell (MSC) chondrogenic differentiation regulation. We objected to examine how miR-218 regulate chondrogenic differentiation of synovium-derived MSCs (SDSCs) and the maturation of RCJ3.1C5.1 chondrocytes. SDSCs were sourced from the knee joint synovium of New Zealand white rabbits, and their multilineage differentiation potentials were examined. The level of miR-218 was measured during SDSC chondrogenic differentiation, together with determination of SDSCs chondrogenic markers and RCJ3.1C5.1 chondrocytes maturation markers expression level after transfection of miR-218 mimics/inhibitor.

**Results:**

miR-218 expression was notably upregulated in early chondrogenesis but mostly ceased during the maturation phases of chondrogenic differentiation in SDSCs. The transfection of miR-218 mimics notably enhanced SDSCs chondrocytes differentiation, as evidenced by augmented expressions of chondrogenic markers (SOX9, COL2A1, ACAN, GAG, and COMP) in terms of mRNA and protein level, and the inhibition of miR-218 yielded opposite resutls. Additionally, miR-218 overexpression substantially suppressed the expression of osteogenic markers (ALP, BSP, COL1A1, OCN and OPN) during the early stage of chondrogenesis while increasing that of chondrogenic markers (SOX9, COL2A1, ACAN, GAG and COMP). However, miR-218 mimics notably suppressed maturation markers (CMP, COL10A1, MMP-13 and VEGF) expression in RCJ3.1C5.18 chondrocytes, and the miR-218 inhibitor promoted the expression of these maturation markers. We proposed miR-218 plays a regulatory role on 15-hydroxyprostaglandin dehydrogenase (HPGD), which plays a key role in chondrogenic differentiation, and this finding indicates that miR-218 directly regulates HPGD expression in SDSCs.

**Conclusion:**

Our study suggests that miR-218 contributes to early chondrogenesis while suppressing later chondrocyte maturation. The miR-218-HPGD pathway offers us a perspective into how SDSCs differentiate into chondrogenic cells.

## Background

Articular cartilage (AC) is an avascular tissue, and thus is characterised with restricted ability to repair itself after trauma or degenerative disease [1]. Various methods are attempted to treat AC injuries, including abrasion under arthroscope, transplantations with mature chondrocytes and mesenchymal stem cells (MSCs), or grafted with tissues like periosteum. Autologous chondrocyte transplantation (ACT), which delivers autologous chondrocytes into the area of defect [2], has been proposed by most clinicians as an feasible approach for cartilage repair, though randomized trials have reported little clinical benefit with this procedure compared with subchondral bone microfracture [3]. An apparent drawback with ACT is it involved morbidity to the donor-site and therefore resultant osteoarthritic changes in the donor joint [4], emphasizing the need for alternative sources of chondrocyte cells. Adult stem cells have been proposed as an excellent cell candidate for cartilage repair due to their proliferation and multilineage differentiation capacity. Especially, synovium-derived MSCs (SDSCs) are unique because of their tissue specificity for cartilage regeneration [5], and these cells have been shown to have great potential in cartilage regeneration research.

MicroRNAs (miRNAs) is a class of small, noncoding, single-stranded RNAs that regulate at posttranscriptional level through either mRNA translation inhibition or mRNA degradation promotion. There are many reports on critical roles miRNAs play in biology, such as differentiation, development, proliferation, and tumorigenesis. Moreover, several miRNAs have been identified to modulate cartilage differentiation and degradation [6–8]. For example, miR-365 targets histone deacetylase 4 and enhances chondrogenesis [9], and miR-145 inhibits early chondrogenesis by targeting Sox9 [10]. Recent reports have found that miR-218 targets the transcription factor Runx2 to control development pathway in osteogenic lineage [11]. It also activates WNT signaling, which play a role in the MSCs chondrogenic differentiation [12, 13]. However, the paticipation of miR-218 in SDSC chondrogenic differentiation and maturation stays unclear.

As an important regulator, prostaglandin E2 (PGE2) significantly promotes chondrogenic markers (SOX9, ACAN, COMP and COL2A1) expression in the early stage of chondrogenic differentiation and inhibits chondrocyte maturation in the late stage. HPGD is a known effecter that regulates PGE2 by converting it to its biologically inactive metabolite. Taken together, Runx2 and HPGD (dehydrogenase of PGE2) play key roles in chondrogenic differentiation and chondrocyte maturation during chondrogenic differentiation.

## Methods

### Isolation and culture of SDSCs

Synovial tissue was harvested from the knees of New Zealand White rabbits which were obtained from the Animal Center of Second Military Medical University. Surgeries were approved by Animal Experimentation Ethics Committee of Second Military Medical University. The rabbits were anaesthetized through the intravenous injection of 0.6% pentobarbital sodium; the injection dosages were 22–26 mg/kg, and the injection rates were 10–20 ml/5–10 min. At a respiratory rate of 46–68/min, the heart rate was 180–210/min, and the corneal reflex was weakened, indicating that the rabbit was under anesthesia. After the isolation of synovial tissue, all the rabbits were alive and fed at the Animal Center of Second Military Medical University. The synovial tissue was finely minced and digested with 0.1% trypsin (Roche DiACANostics, Mannheim, Germany) at 37 °C for 30 min and then with 0.1% collagenase D (Roche) for 2 h. Cells were collected after filtering and plated at clonal density in 60-cm^2^ culture dishes (Nalge Nunc International, Rochester, N.Y., USA) in complete medium (α-minimum essential medium containing 10% fetal bovine serum, 100 U/mL penicillin, 100 mg/mL streptomycin, and 0.25 mg/mL fungizone [Invitrogen, Carlsbad, CA]) and incubated at 37 °C with 5% humidified CO_2_. Non-adherent cells were removed when the medium was changed every two days. SDSCs were harvested when adherent cells reached ~ 80% confluence.

### Detection of SDSC surface markers

A flow cytometry analysis was performed to confirm isolated SDSCs surface markers as previously described [14]. Anti-rabbit CD44-PE, CD90-FITC (Abcam, Cambridge, MA, USA), CD105-FITC, CD14-FITC, CD34-FITC, and CD45-FITC (eBioscience, San Diego, CA, USA) were employed as monoclonal antibodies. Flow cytometry was conducted with a FACSCalibur cytometer (BD Biosciences), and data analyses were performed using BD FAC Suite software (BD Biosciences).

### Adipogenic, osteogenic and SDSCs chondrogenic differentiation

SDSCs at passage 3 were collected and seeded in a six-well plate at a density of 1 × 10^5^/well. When they reached ~ 80% confluence, the SDSCs were incubated in either adipogenic differentiation medium, osteogenic differentiation medium, or chondrogenic differentiation medium supplemented with 10 ng/mL transforming growth factor beta 3 (TGF-β3). All three types of differentiation media were purchased from BD Biosciences (Bedford, MA, USA), and the culture medium was changed every 3 days. Staining for adipogenic, osteogenic, or chondrogenic differentiation was performed with fresh Oil Red O solution (Sigma-Aldrich, MO, USA) for 2 h, 0.1% Alizarin Red (Sigma-Aldrich, MO, USA) solution for 40 min, or 1% Alcian Blue solution (Sigma-Aldrich) for 30 min at room temperature, respectively.

### Lentivirus-mediated cell transfection

Recombinant lentivirus miR-218-mimics and miR-218-inhibitor were purchased from GeneChem (Shanghai, China). For the lentivirus infection, differentiated SDSCs and RCJ3.1C5.18 chondrocytes were seeded in a 12-well plate and cultured overnight to 70% confluence according to the manufacturers’ instructions. The cells were then transfected with different lentiviruses for 24 h.

### Reverse transcription quantitative polymerase chain reaction (RT-qPCR)

Total RNA was extracted using TRIzol reagent (Life Technologies Inc., Grand Island, NY) and RNeasy Mini Kit (Qiagen, Valencia, CA) according to the manufacturer’s instructions. Approximately 1 μg of mRNA was used for reverse transcriptase with a High-Capacity cDNA Archive Kit (Applied Biosystems) at 37 °C for 2 h. For miRNA detection, reverse transcription was performed using miRNA-specific stem-loop primers, and real-time PCR was performed using SYBR green Master Mix. Chondrogenic marker genes *SOX9, COL2A1, ACAN, GAG* and *COMP*, osteogenic marker genes *ALP, BSP, Col1a1, OCN* and *OPN*, and maturation markers *CMP, Col10a1, MMP-13*, and *VEGF* were customized by Applied Biosystems as part of Custom TaqMan Gene Expression Assays (Table 1). β-Actin and U6 were employed as internal controls to determine mRNA and miRNA expression levels. The RT-PCR conditions were as follows: an initial 10-min incubation at 95 °C, 40 cycles of 95 °C for 10 s, 60 °C for 20 s and 72 °C for 30 s, and 5 min at 4 °C. Relative quantification analysis was conducted using the 2^−△△CT^ method. Each sample was analyzed in triplicate, and all experiments were carried out three times independently.

**Table 1:** Sequences of primers used for real-time PCR analysis

Name	Forward primer (5’-3’)	Reverse primer (5’-3’)
Chondrogenic markers		
Sox9	TGGCAAGGCTGACCTGAAG	GCTCAGCTCGCCGATGT
Col2a1	TCCTGGCCTCGTGGGT	GGGATCCGGGAGAGCCA
Aggrecan	GCCACTGTTACCGCCACTT	CACTGGCTCTCTGCATCCA
COMP(cartilage oligomeric matrix protein)	ACTGCCTGCGTTCTAGTGC	CGCCGCATTAGTCTCCTGAA
Osteogenic markers		
ALP	CCCTTCACTGCCATCCTGTAC	CCATGGAGACGTTCTCTCTCTCA
BSP (bone sialoprotein)	ATGCCTGCCTTGTACCACGAGC	TCCATCGAAGAATCAAAGCAGAG
Col1a1	CCGTGCCCTGCCAGATC	CAGTTCTTGATTTCGTCGCAGATC
OCN(Osteocalcin)	GGCAGGGAAGTCAGGGTAG	CCCGTGGTTTCCTGGTC
OPN(Osteopontin)	GTACCCTGATGCTACAGACG	TTCATAACTGTCCTTCCCAC
Mature markers		
CMP(cartilage matrix protein)	AAGAACGACGACCAAAAGGAC	CATCCCCTATACCATCGCCA
Col10a1	GGCCCGGCAGGTCATC	TGGGATGCCTTTTGGTCCTT
MMP-13	AGTTTGGCCATTCCTTAGGTCTTG	GGCTTTTGCCAGTGTAGGTATAGAT
VEGF	GGCTGGCAACATAACAGAGAA	CCCCACATCTATACACACCTCC
β-actin	CTGAACCCTAAGGCCAACCG	GTCACGCACGATTTCCCTCTC

### Western blot analysis

For total protein extraction, cells were lysed in RIPA buffer (GuHCl, Sigma-Aldrich) supplemented with phenylmethanesulfonyl fluoride (PMSF) and centrifuged at 12,000×g and 4 °C for 10 min. The supernatant was collected, and total protein was quantified using a BCA Protein Assay Kit (Sigma-Aldrich) according to the manufacturer’s protocols. Equal amounts (40 μg) of protein samples were separated by 10% sodium dodecyl sulfate-polyacrylamide gel electrophoresis (SDS-PAGE) and transferred onto polyvinylidene fluoride (PVDF) membranes (Millipore, Billerica, MA, USA). After blocking with 5% nonfat milk in TBST (100 mM Tris-HCl, 0.9% NaCl, 1% Tween 20, pH 7.5) for 1 h, membranes were individually incubated with primary monoclonal antibodies against chondrogenic markers SOX9, COL2A1, ACAN, GAG and COMP, osteogenic markers ALP, BPS, COL1A1, OCN and OPN, and maturation markers CMP, COL10A1, MMP-13 and VEGF in 1% nonfat milk in TBST for 3 h at room temperature. Membranes were then incubated with a secondary horseradish peroxidase (HRP)-conjugated goat anti-rabbit antibody (dilution factor of 1:5000) for 40 min at room temperature, followed by exposure in SuperSignal West Femto Maximum Sensitivity Substrate using CL-XPosure Film.

### Immunocytochemistry

A total of 2.5 × 10^5^ passage-3 SDSCs were seeded into 15-mL centrifuge tubes and cultured in 0.5 mL of chondrogenic differentiation medium for 21 days. The pellets (*n* = 2) were fixed in 4% paraformaldehyde at 4 °C overnight, followed by dehydration in a gradient ethanol series, clearing with xylene, and embedding in paraffin blocks. Sections with a thickness of approximately 5 mm were stained with Alcian Blue (Sigma; counterstained with Fast Red) and Safranin O (Sigma; counterstained with hematoxylin) for sulfated glycosaminoglycans (GAGs). For immunohistochemistry analysis, sections were probed with primary antibodies against collagen II (II-II6B3; DSHB, Iowa City, IA, USA), followed by the secondary antibody biotinylated horse anti-rabbit IgG (Vector, Burlingame, CA, USA). Immunoactivity was detected using Vectastain ABC reagent (Vector) with 3,3′-diaminobenzidine (DAB) as a substrate. Hematoxylin (Vector) served as a counterstain.

### Luciferase reporter assay

pMiR-REPORT-mut-HPGD 3’UTR constructs containing the HPGD 3′ untranslated region (UTR) with three point mutations in the seed sequence were produced using a QuikChange II Site-Directed Mutagenesis kit (Stratagene). HEK293 cells were seeded in a 96-well plate for 24 h and then cotransfected with pMIR-REPORT-HPGD 3’UTR plasmid or pMIR-REPORT-mut-HPGD 3’UTR plasmid, internal control pRL-TK-Renilla-luciferase plasmid and the indicated RNAs. In contrast, the pMIR-REPORT-HPGD 3’UTR plasmid or pMIR-REPORT-mut-HPGD 3’UTR plasmid and pRL-TK plasmid were transfected into SDSCs in 24-well plates. After the induction of chondrogenic differentiation for 14 days, luciferase activities were determined using a Dual Luciferase Reporter Assay Kit (Promega). All transfection experiments were conducted in triplicate and repeated three times independently.

### Statistical analysis

The experimental values are presented as the means ± standard deviations (SDs). The statistical analyses were conducted with GraphPad Prism Software Version 6.0 (GraphPad Software Inc., La Jolla, CA, USA). Significant differences between groups were evaluated by one-way ANOVA. P<0.05 was considered statistically significant.

## Results

### Identificaiton and determination of multilineage differentiation capacity of SDSCs

**Fig. 1:**
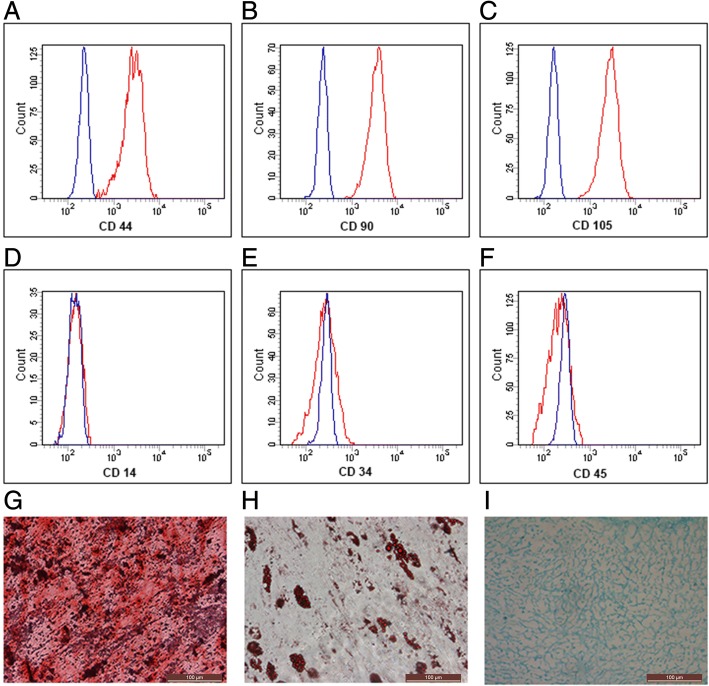
Characterization of MSC properties (**a**-**f**) and multilineage differentiation capacity (**g**-**i**) of SDSCs. Flow cytometry was used to characterize MSC surface phenotypic markers CD44 (**a**), CD90 (**b**), CD105 (**c**), CD14 (**d**), CD34 (**e**), and CD45 (**f**) in SDSCs. Adipogenic, osteogenic and chondrogenic differentiation were assessed using Oil Red O staining (**g**), alkaline phosphatase positivity (**h**) and Alcian Blue staining (**i**), respectively. Bar 100 μm

To confirm whether our cultured SDSCs exhibit the characteristics of MSCs, passage-3 cells were analyzed by flow cytometry. Rabbit SDSCs were positive for CD44, CD90, and CD105 but negative for CD14, CD34 and CD45 (Fig. 1). To further identify the multilineage differentiation potentials of SDSCs, adipogenic, osteogenic or chondrogenic differentiation was induced in the third-passage cells. Following induction for 21 days, SDSCs were positive for Oil Red O staining, alkaline phosphatase or Alcian Blue staining (Fig. 1).

### miR-218 is upregulated in early chondrogenesis but downregulated in late chondrogenesis in SDSCs

**Fig. 2:**
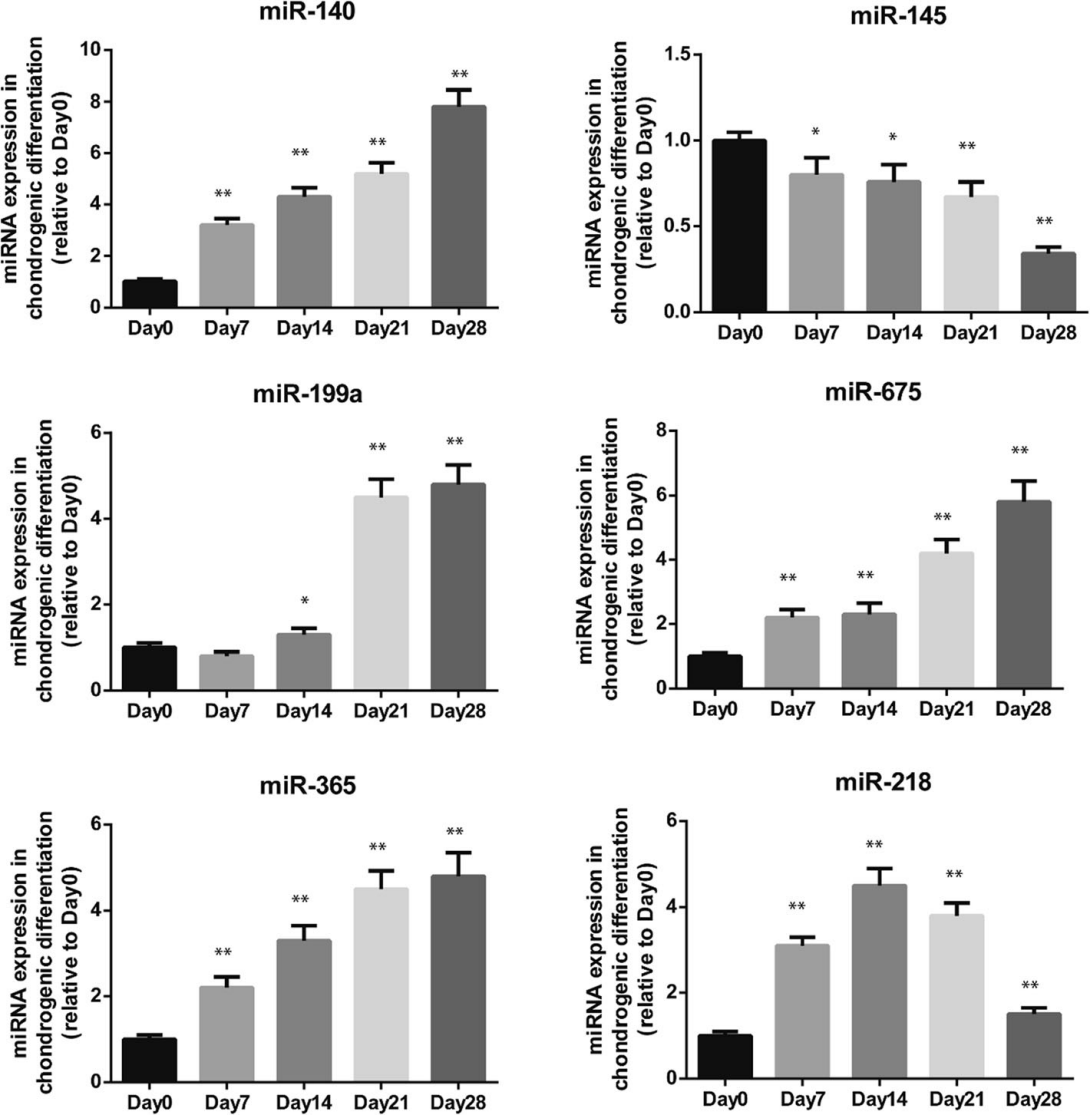
miR-218 was upregulated in SDSCs early in chondrogenesis but downregulated in later chondrogenesis. Expression of 6 miRNAs, miR-140, miR-199a, miR-365, miR-675, miR-145 and miR-218, was determined using quantitative real-time PCR on days 0, 7, 14, 21 and 28 of chondrogenic differentiation. The expression levels of miRNA were normalized to those of U6. The data are presented as the means ± SDs (*n* = 3). *P < 0.05 vs. Day 0, **P < 00.01 vs. Day 0

Expression of 6 miRNAs implicated in chondrogenesis was evaluated by RT-qPCR at 0, 7, 14, 21 and 28 days of chondrogenic differentiation [6–10]. Our results showed that expression of chondrogenesis-related miRNAs miR-140, miR-199a, miR-365, and miR-675 increased gradually during the entire chondrogenesis process, whereas miR-145 levels gradually decreased. Of note, miR-218 increased significantly in a time-dependent manner during early chondrogenesis and peaked at 14 days, though expression of miR-218 was downregulated in late stages of SDSC chondrogenesis (Fig. 2).

### miR-218 enhances SDSCs chondrogenic differentiation during the early stage

**Fig. 3:**
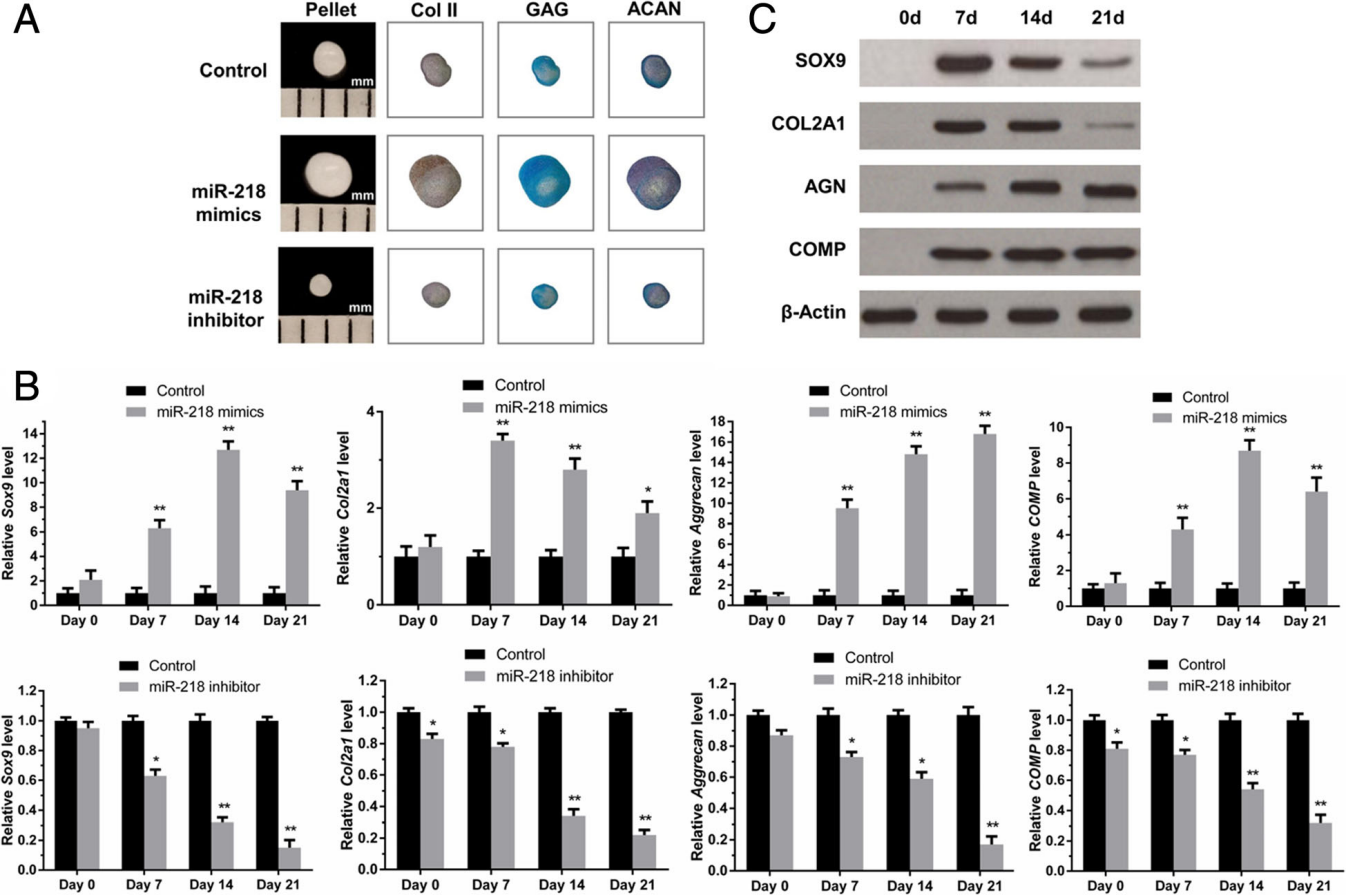
miR-218 promotes SDSC chondrogenesis. SDSCs were transfected with either miR-218 mimics or miR-218 inhibitor. After induction of chondrogenic differentiation for 21 days, (**a**) immunohistochemistry was used to detect Col II, and Alcian Blue and Safranin O were utilized to stain sulfated GAG or ACAN, respectively. **b** RT-PCR was used to measure expression of chondrogenic marker genes, including *SOX9, COL2A1, Aggrecan*, and *COMP*, in miR-218-overexpressing and miR-218-inhibited SDSCs. Three independent experiments were performed in triplicate, and the data are presented as the means ± SDs. * *P* < 0.05 vs. the control group, ** *P* < 0.01 vs. the control group. **c** miR-218 mimics were transfected into SDSCs, and SOX9, COL2A1, Aggrecan and COMP protein levels were assessed by western blot analysis

Twenty-one days after induction of chondrogenic differentiation, immunohistochemistry staining was employed to detect type II collagen (Col II); Alcian Blue and Safranin O were used to stain sulfated glycosaminoglycan (GAG) and aggrecan (ACAN). The mRNA and protein levels of SOX9*,* COL2A1*,* ACAN and COMP in SDSCs were measured at 0, 7, 14 or 21 days of chondrogenic differentiation. As shown in Fig. 3a, the size of the cell pellet increased with miR-218 mimic transfection but decreased following miR-218 inhibitor transfection. Additionally, RT-qPCR results showed that miR-218 mimic transfection led to a significant increase in chondrogenic marker mRNA (Fig. 3b) and protein expression levels (Fig. 3c), whereas these chondrogenic markers were markedly downregulated in miR-218 inhibitor-transfected SDSCs.

### miR-218 represses early osteogenesis of SDSCs

**Fig. 4:**
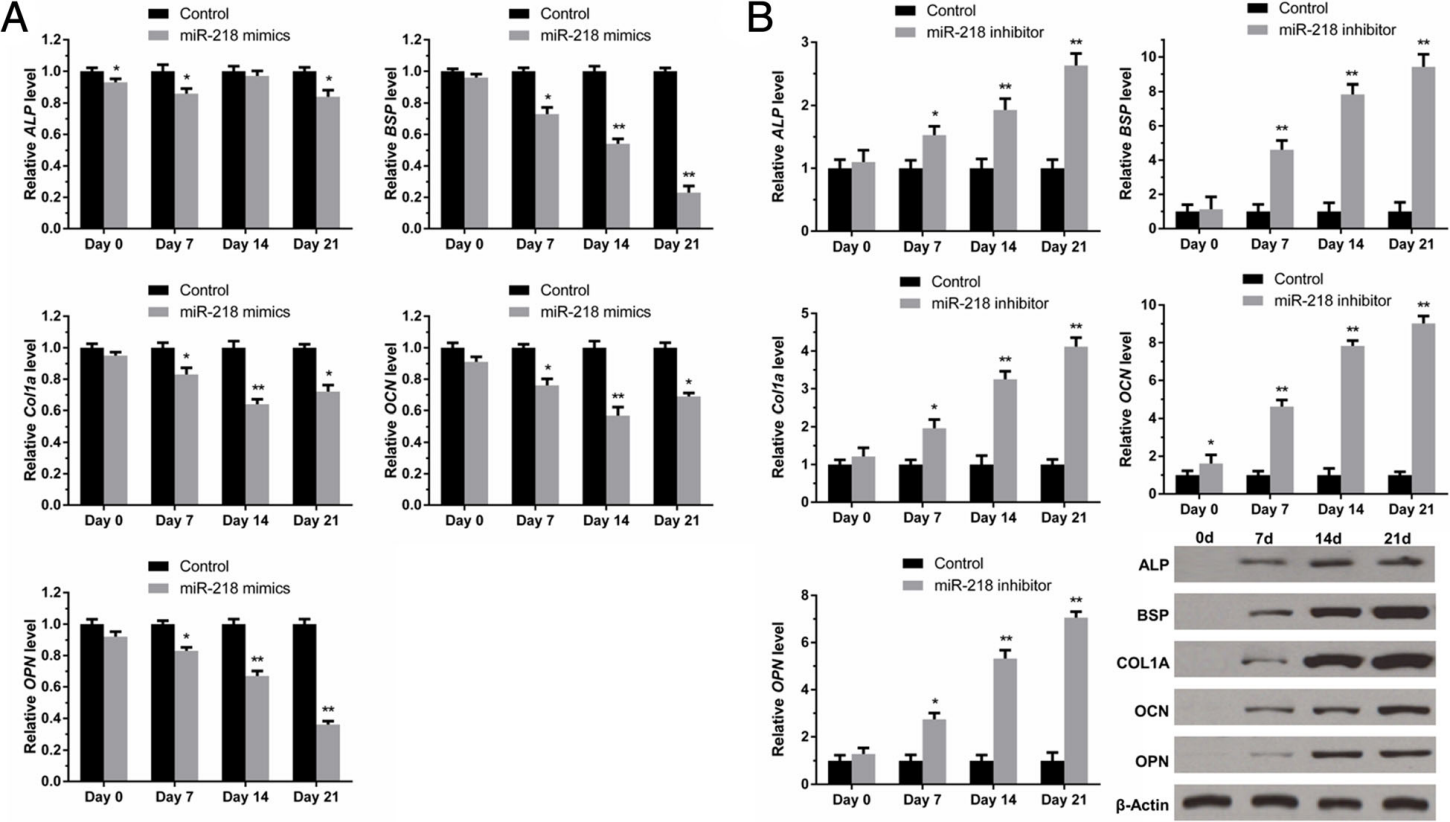
miR-218 represses SDSC osteogenesis. SDSCs were transfected with either miR-218 mimics or miR-218 inhibitor. **a** After induction of chondrogenic differentiation for 21 days, expression of osteogenic marker genes, including *ALP, BPS, Col1a, OCN*, and *OPN*, was measured by RT-PCR in miR-218-overexpressing SDSCs. Three independent experiments were performed in triplicate, and the data are presented as the means ± SDs. * *P* < 0.05 vs. the control group, ** *P* < 0.01 vs. the control group. P (**b**) miR-218 inhibitor was transfected into SDSCs, and ALP, BPS, COL1A1, OCN and OPN protein levels were measured by western blot analysis. * *P* < 0.05 vs. the control group, ** *P* < 0.01 vs. the control group

Twenty-one days after induction of chondrogenic differentiation, the mRNA and protein levels of osteogenic markers ALP, BSP, Col1a1, OCN, and OPN were examined at days 0, 7, 14 and 21. As shown in Fig. 4a, RT-qPCR results showed that miR-218 mimic transfection led to a significant decrease in osteogenic marker mRNA expression levels; in contrast, these osteogenic markers were markedly upregulated in miR-218 inhibitor-transfected SDSCs, and western blotting results were consistent with the results of RT-qPCR (Fig. 4b).

### miR-218 inhibits chondrocyte maturation

**Fig. 5:**
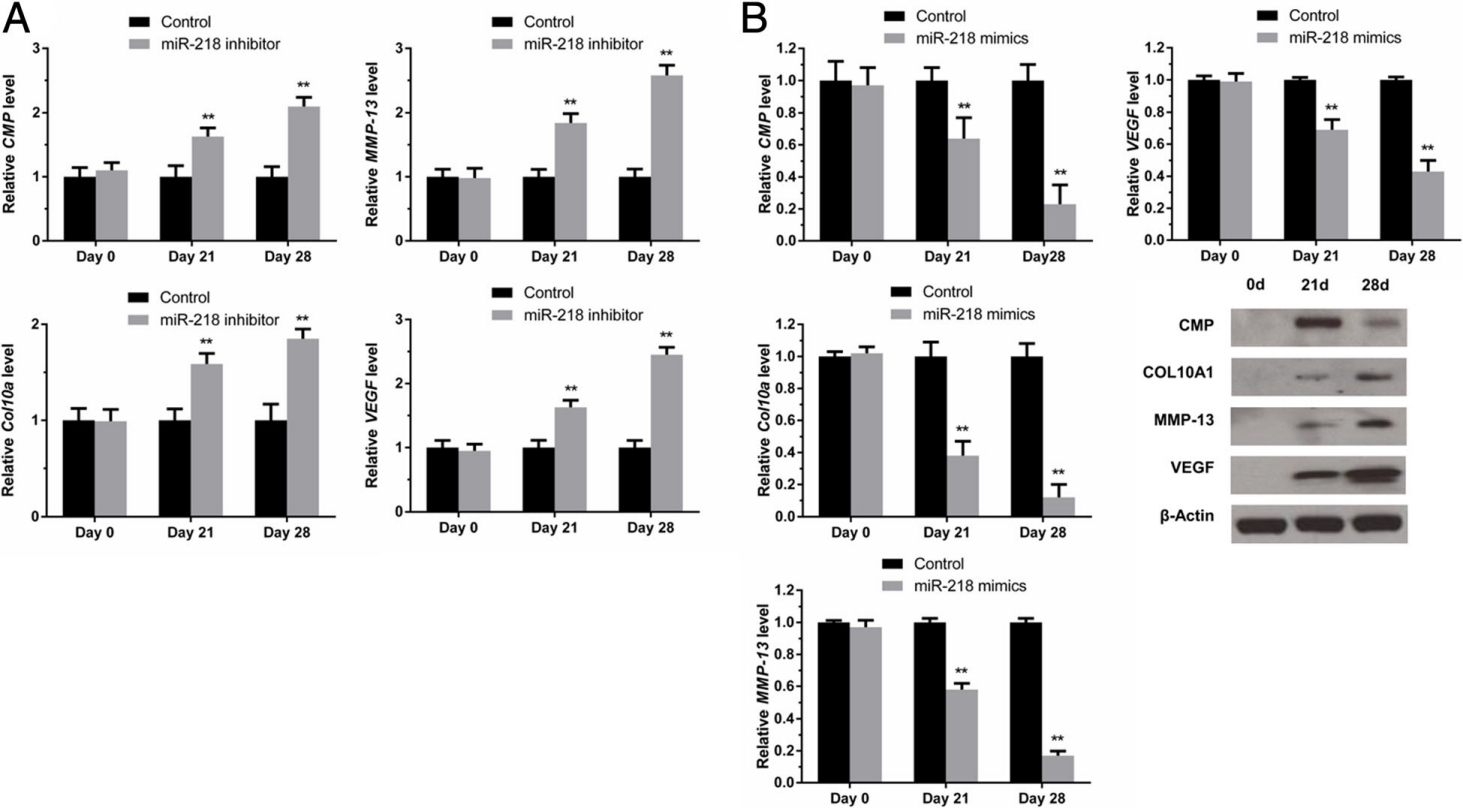
miR-218 inhibits chondrocyte maturation. C5.18 chondrocyte cells were transfected with either miR-218 mimics or miR-218 inhibitor. **a** After induction of chondrogenic differentiation for 28 days, expression of mature marker genes, including *CMP, Col10a1, MMP-13* and *VEGF*, was measured by RT-PCR in miR-218-overexpressing C5.18 chondrocyte cells. Three independent experiments were performed in triplicate, and the data are presented as the means ± SDs. ** *P* < 0.01 vs. the control group. **b** miR-218 inhibitor was transfected into C5.18 chondrocyte cells, and CMP, COL10A1, MMP-13 and VEGF protein levels were measured by western blot analysis. ** *P* < 0.01 vs. the control group

Twenty-eight days after induction of chondrogenic differentiation, the mRNA and protein levels of the maturation markers CMP, COL10A1, MMP-13, and VEGF were examined at days 0, 21 and 28. RT-qPCR revealed that miR-218 mimic transfection led to a significant decrease in mRNA expression of maturation marker, whereas levels of these markers were markedly increased in miR-218 inhibitor-transfected C5.18 chondrocytes (Fig. 5a). Western blotting results were consistent with the RT-qPCR results (Fig. 5b).

### miR-218 directly regulates HPGD expression in SDSCs

**Fig. 6:**
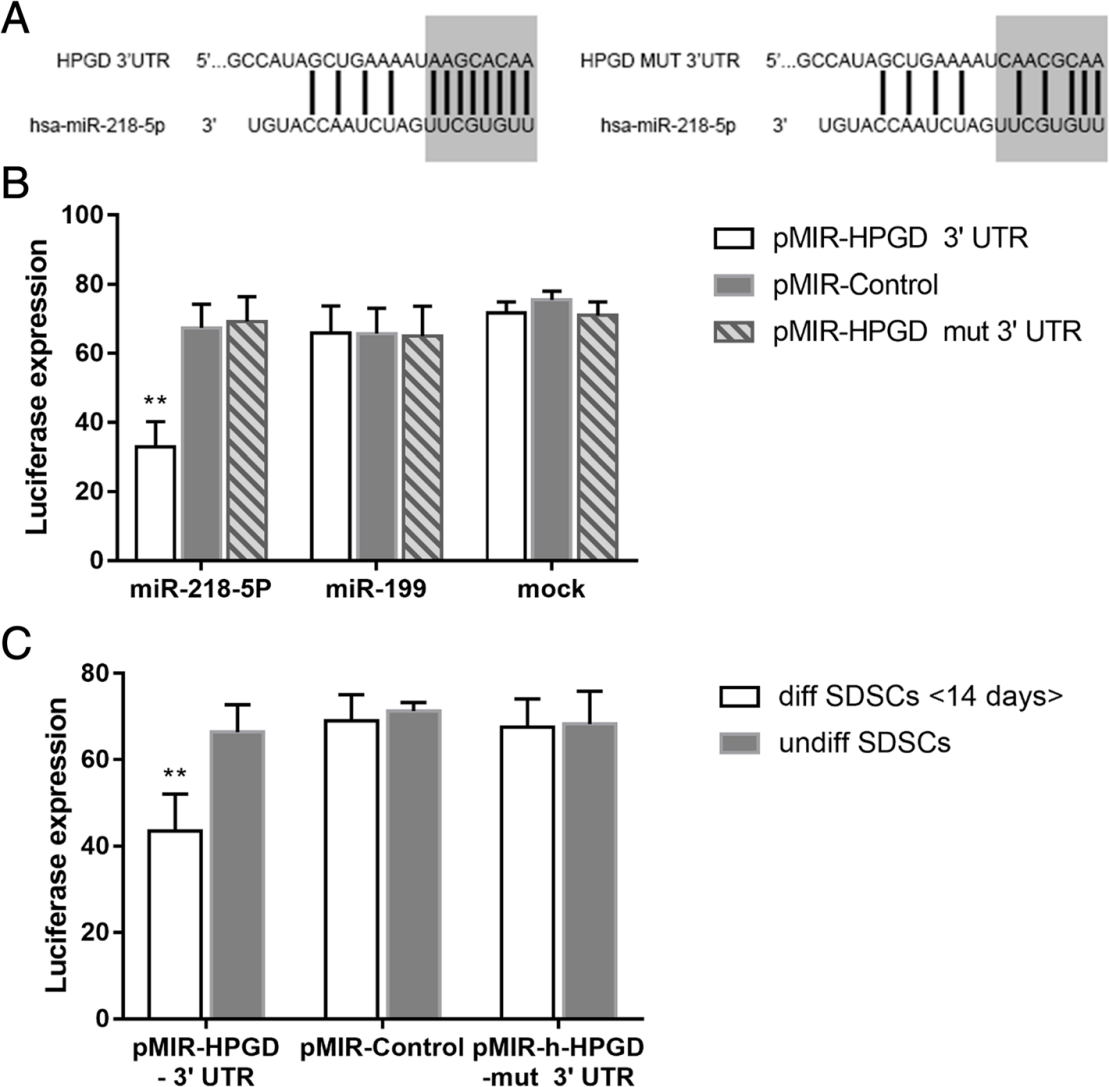
miR-218 directly regulates HPGD expression in SDSCs. The predicted pairing of miRNAs and their target regions in the wild-type HPGD 3’UTR or the mutant (mut) HPGD 3’UTR are shown (**a**). HEK293 cells were cotransfected with the firefly luciferase-expressing vector pMIR-REPORT containing the wild-type HPGD 3’UTR, the mut HPGD 3’UTR or the control insert as well as the internal control Renilla-luciferase-expressing vector pRL-TK and the indicated RNAs. miR-199 was cotransfected with reporters as a negative RNA control. Luciferase activities were measured after 48 h. The data were normalized by dividing firefly luciferase activity with that of Renilla luciferase; **, *P* < 0.01 (**b**). Luciferase activities were measured in SDSCs cotransfected with the pMIR-REPORT plasmid containing wild-type HPGD 3’UTR, mut HPGD 3’UTR or control insert and the internal control pRL-TK. All samples were assayed in duplicate (*n* = 3). **, *P* < 0.01 (**c**)

The TargetScan bioinformatics tool was used to search for miRNAs targeting the HPGD 3’UTR, and only miR-218-5p and miR-26-5p were predicted as candidate miRNAs. Moreover, miR-218-5p was predicted to target two conserved regions of HPGD. Luciferase reporters carrying either the wild-type 3’UTR of HPGD or a mutant 3’UTR with three point mutations in the target site (as a negative control) were engineered to investigate whether HPGD is directly targeted by miR-218 (Fig. 6a) and were then cotransfected with miR-218 mimics into HEK293 cells. Compared with the negative control, the luciferase activity of the wild-type HPGD reporter were significantly reduced by miR-218 mimics. miR-199 was also cotransfected with reporters as a negative RNA control. In contrast, the mimics of miR-218 did not repress the expression of mutant reporters (Fig. 6b). To examine the role of endogenous miR-218 in inhibiting the HPGD 3’UTR reporter in chondrogenic differentiation, SDSCs were cotransfected with the wild-type 3’UTR luciferase reporter and the luciferase reporter with the mutant UTR as a negative control at 14 days after chondrogenic differentiation, and significant inhibition of the wild-type HPGD 3’UTR luciferase reporter activity was observed in the differentiated compared with undifferentiated SDSCs (Fig. 6c). Taken together, these results show that miR-218 directly regulates HPGD by targeting its 3’UTR and that miR-218 might play a prominent role during chondrogenic differentiation.

## Discussion

Because miRNAs are central to the regulations of self-renewal and differentiation of stem cells [15, 16], stem cells represent novel targets for regulating their behavior and differentiation. In this study, we have examined how miR-218 regulates SDSCs chondrogenic differentiation and RCJ3.1C5.1 chondrocytes maturation. miR-218 was found to be dramatically upregulated in SDSC early chondrogenesis but was downregulated in late stages. Moreover, miR-218 mimics strongly promoted the differentiation of SDSCs into chondrocytes. In contrast, miR-218 inhibition suppressed SDSC differentiation into chondrocytes. In addition, the level of osteogenic marker expression was found to significantly decrease in the early stage of chondrogenesis, and miR-218 overexpression might negatively regulate this process. Chondrogenic markers were dramatically increased by overexpression of miR-218. Furthermore, transfection of miR-218 mimics markedly suppressed expression of maturation markers in RCJ3.1C5.18 chondrocytes, but miR-218 inhibitor notably promoted expression of these maturation markers. miR-218 might directly regulate HPGD expression in SDSCs. These findings indicate that miR-218 possibly regulates SDSC chondrogenesis via the miR-218-HPGD pathway.

Articular cartilage is a unique tissue characterized by low cellular density and a lack of vascular components. The major composition of articular cartilage is proteoglycans and extracellular collagens and it has a very limited self-healing capacity in pathological conditions such as injury and degeneration [1]. Moreover, treatment options for cartilage trauma have dramatic differences in terms of effectiveness. Although early clinical studies indicated the effectiveness of ACT [17], randomized studies have reported little value of this ACT procedure in terms of effectiveness when compared with inducing subchondral bone microfracture [3]. ACT also involves donor-site morbidity and joint osteoarthritic alterations [4]. Therefore, alternative cell sources are required. MSCs have been recommended as candidates because they are capable of differentiating into chondrocytes with biological functions. Additionally, the similarity between differentiated MSCs and host cells render them ideal for cartilage replacement therapy [18]. Accordingly, transplantation of MSCs possessing chronic differentiation capacity might be an effective treatment strategy for articular cartilage injury. However, despite successful reports, MSC transplantation has also been associated with drawbacks; for example, transplantation of MSCs may lead to heterogeneous undifferentiated and differentiated populations [19]. Thus, new strategies are needed to enhance dedicated chondrocytes differentiation from MSCs.

There are abundant experimental findings pointing at the critical regulatory role that miRNAs play in MSC chondrogenic differentiation. By suppression of certain genes, several miRNAs have been shown to regulate chondrocytes differentiation from MSCs. For instance, miR-140 [20], miR-23b [21], miR-455 [20], and miR-335 [22] were found to boost chondrogenic differentiation by suppressing *ADAMTS5*, *PRKACB*, *Smad2/3*, and *ROCK1*. On the other hand, miR-574 [23], miR-29a [24], and miR-495 [25] were found to downregulate chondrogenesis by inhibiting *RXRα*, *FOXO3A*, and *SOX9*. WNT signaling plays an important role in the MSCs chondrogenic differentiation [13], and miR-218 activates WNT signaling pathway by targeting its negative regulatory factors [12]. Based on these results, we hypothesized that miR-218 might positively regulate MSC chondrogenic differentiation as well as maturation of RCJ3.1C5.1 chondrocytes. Our findings clarified that miR-218 acts as a promoter during early chondrogenic differentiation of SDSCs and as a silencer during later chondrocyte maturation.

Chondrogenesis has been divided into two phases: early chondrogenesis and hypertrophy. The former stage is featured by upregulated *COL2A1* and *SOX9*, and the latter stage is evidenced by upregulated *Col10a1* and *Runx2* [26]. Both phases are inter-regulated via interactions among several signaling pathways [27], and they antagonize each other [28–31]. Runx2, a key regulatory gene in osteogenic differentiation, mediates many osteogenic-related genes. However, Runx2 suppresses MSCs chondrogenic differentiation in the early stage and promotes later chondrocyte maturation [32]. PGE2 may promote cell proliferation and increase *SOX9*, *COL2A1*, and *aggrecan* expression in the early phase of MSCs chondrogenic differentiation while restraining later chondrocyte maturation. These findings reveal that Runx2 and PGE2 may play critical roles in early chondrogenic differentiation and later chondrocyte maturation.

In conclusion, our results showed significant upregulation of miR-218 early in SDSC chondrogenic differentiation and downregulation later during chondrocyte maturation. miR-218 overexpression enhances expression of chondrogenic markers, promoting early SDSC chondrogenic differentiation and suppressing later chondrocyte maturation. Moreover, miR-218 may regulate SDSC chondrogenesis via the miR-218-HPGD pathway. Therefore, miR-218 mimics may constitute a therapeutic strategy when applying SDSC-based therapy for the treatment of cartilage-related disorders.

## Conclusion

Our study suggests that miR-218 contributes to early chondrogenesis while suppressing later chondrocyte maturation. The miR-218-HPGD pathway offers us a perspective into how SDSCs differentiate into chondrogenic cells and constitute a therapeutic strategy when applying SDSC-based therapy for the treatment of cartilage-related disorders.

## Abbreviations

AC: Articular cartilage
ALP: Alkaline phosphatase
COMP: Cartilage oligomeric matrix protein
GAGs: Glycosaminoglycans
HPGD: 15-Hydroxyprostaglandin dehydrogenase
MMP-13: Metal matrix proteinase 13
MSC: Mesenchymal stem cell.
OCN: Osteocalcin
OPN: Osteopontin
PBS: Phosphate-buffered saline
PGE2: Prostaglandin E2
Runx2: Runt-related transcription factor 2
SDs: Standard deviations
SDSCs: Synovium-derived MSCs
SOX9: SRY-related gene 9
TGF-β3: Transforming growth factor beta 3
VEGF: Vascular endothelial growth factor
